# Genome-wide analysis of *DGAT* gene family in *Coix lacryma jobi* L. and functional characterization in yeast H1246

**DOI:** 10.1186/s12870-025-07648-7

**Published:** 2025-11-28

**Authors:** Tinghui Feng, Jiacong Gao, Yuan Li, Lijun Yang, Xiaodan Zhang, Juane Dong, Zongsuo Liang

**Affiliations:** 1 College of Life Sciences, Northwest A & F University, Xi’an, 710000 China; 2 College of Life Sciences and Medicine, Zhejiang Sci-Tech University, Hangzhou, 310018 China

**Keywords:** *Coix lacryma jobi L.*, *Diacylglycerol acyltransferase* (*DGAT*), Gene family, Yeast H1246, Oil content, Fatty acids content, Thin-layer chromatography (TLC) test, Nile red staining

## Abstract

**Background:**

*Coix lacryma jobi* L., a member of the *Poaceae* family, is a traditional Chinese medicine ingredient with a long history. In recent years, research and clinical treatment have shown that *Coix lacryma jobi* L. seed oil exhibits significant anti-cancer effects and enhances the efficacy of chemotherapy. As *Diacylglycerol Acyltransferase* is a key rate-limiting enzyme in lipid synthesis, a thorough understanding of the *Diacylglycerol Acyltransferase* gene family in *Coix lacryma jobi* L. could pave the way for increasing its lipid content.

**Results:**

This study used the website NCBI to search for the CDS sequences of the *Diacylglycerol Acyltransferase* family genes in different plants and find the homologous genes in *Coix lacryma jobi* L. genome by blast screening. A total of ten *Diacylglycerol Acyltransferase* family genes were identified in *Coix lacryma jobi* L., which were named *ClDGAT1_1, ClDGAT1_2*, *ClDGAT1_3*, *ClDGAT2_1*, *ClDGAT2-2*, *ClDGAT3*, *ClWS/DGAT_1*, *ClWS/DGAT_2*, *ClWS/DGAT_3*, and *ClWS/DGAT_4*. We analyzed the protein physicochemical properties, gene structure, gene homology and evolutionary analysis of them to elaborate the information of *ClDGAT* family genes. Meanwhile, functional assays revealed significant differences in oil and fatty acid synthesis among the *ClDGAT*s. By expressing ten *ClDGAT* genes in H1246 yeast and comparing the differences in oil and fatty acid content in these yeasts, we found that *ClDGAT3* and *ClDGAT1_2* had the best oil and triglyceride synthesis ability. This study advanced research on the *Diacylglycerol Acyltransferase* gene family and expanded the understanding of lipid synthesis-related genes in *Coix lacryma jobi* L..

**Conclusions:**

In this study, we systematically identified and characterized ten *DGAT* family genes in the *Coix lacryma jobi* L. genome. Functional validation in the H1246 yeast demonstrated significant divergence in lipid synthesis capacity of *ClDGAT* isoforms. And *ClDGAT3* and *ClDGAT1_2* had the best oil and triglyceride synthesis ability.

**Supplementary Information:**

The online version contains supplementary material available at 10.1186/s12870-025-07648-7.

## Background

*Coix lacryma jobi* L., a member of the *Gramineae family*, is also known as Job's tears, grain rice, medicine king rice, etc. Because of its high nutritional value, it is known as the "king of *Gramineae*" [1, 4]. It is one of Chinese important food cultivars, with homologous of medicine and food [4, 12]. It flowers and fruits in July to October or in June to December [15, 20]. It is recorded in the ‘Compendium of Materia Medica’ that *C. lacryma-jobi* L. has the efficacy of treating contractures, strengthening the spleen, tonifying the lungs, clearing heat, and removing dampness, etc. The modern pharmacological research has shown that *C. lacryma-jobi* L. has multiple pharmacological effects, such as anti-tumor, hypoglycemia, anti-inflammation, analgesic, immune regulation and regulation of lipid metabolism, etc. [8, 29, 32, 41].

The oil extracted from *Coix* seed has an important value of anti-cancer [23, 29, 39]. It has been widely used in the treatment and adjuvant therapy of various diseases, such as lung adenocarcinoma, colorectal cancer, cervical cancer, lymphoma, gastric cancer, cholangiocarcinoma, glioma, lung cancer, and liver cancer [3, 13, 29]. Because of the anticancer activity of *Coix* oil, it has been used in clinical treatment. For example, Kanglaite Injection (KLTi, National Medical License No.: Z14021231) is mainly composed of coix seed oil. It has been clinically used to treat a variety of cancers, including non-small cell lung cancer, gastric cancer, pancreatic cancer, and so on [17, 22, 43].

**Fig. 1:**
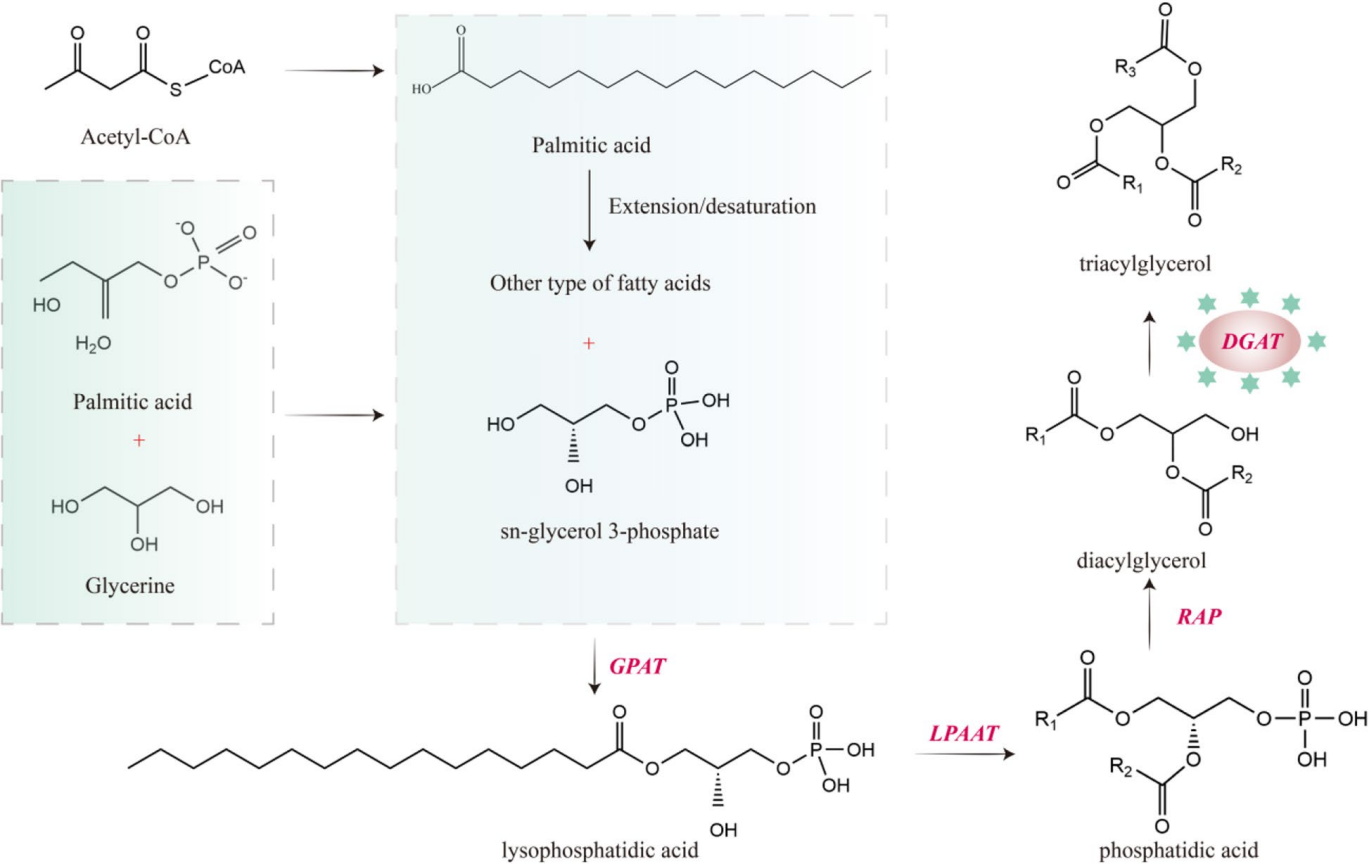
The TAG biosynthesis pathway. *GPAT*: Glycerol-3-phosphate acyltransferase, *LPAAT*: LPA acyltransferase; *DGAT*: Diacylglycerol Acyltransferase

Triacylglycerol (TAG) is the main storage form of plant seed lipids. The TAG biosynthesis pathway is distinguished into the Kennedy pathway and the non-dependent lipoyl CoA pathway (PDAT pathway). In the Kennedy pathway, Glycerol-3-phosphate acyltransferase (*GPAT*), LPA acyltransferase (*LPAAT*), and Diacylglycerol Acyltransferase (*DGAT*) catalyzes the binding of 3-phosphoglycerol sn-1, sn-2, and sn-3 positions to the lipoyl group, respectively, with *DGAT* being the key rate-limiting enzyme in this pathway [21, 44]. Figure 1 summarized the TAG synthesis pathway and the enzymes which are required for each reaction. It can be seen that *DGAT*, as the key enzyme in the final step, directly determines the efficiency of triglyceride synthesis. Several studies have shown that *DGAT* plays a role in the synthesis and accumulation of lipids in many plants [27, 33]. For example, Jing et al. 14 found overexpression of *GmDGAT2A* driven by a seed-specific promoter of *Gmole1* in soybean significantly increased total oil content and linoleic acid content specifically. Han, et al. 10 suggested that *PrDGAT3* may have practical applications in improving plant lipid nutrition and increasing oil production in plants. Xu, et al. 34 conducted oleic acid (18:1) content and total fatty acid content of T_3_ *AhDGAT3* transgenic soybean were significantly higher than those of the wild type (WT). Moreover, a lot of studies found that different *DGAT*s vary widely in function [11, 18]. Therefore, this study hypothesized that the efficiency of acyl transfer catalyzed by different *DGAT* enzymes might be different. However, there have been no reports on *DGATs* in *C. lacryma-jobi* L. (*ClDGAT*). A deeper understanding of the structure and function of the *ClDGAT* family genes will open up new avenues for increasing the oil content in coix seeds. Identifying the most efficient *DGAT* enzyme has great significance for future research on the accumulation of oil in coix seeds, as it can be used as a parent for *DGAT* enzyme gene modification or for constructing stable and efficient *DGAT* overexpression plants.

H1246 yeast is a TAG-deficient yeast mutant strain that has been knocked out of the *DGA1* (encoding DGAT), *LRO1* (encoding PDAT), and *ARE1* and *ARE2* (encoding cholesterol lipid synthesis) genes, which regulate TAG synthesis, and is useful in characterizing the TAG synthesis pathway efficiency. In recent years, H1246 yeast has been extensively used as an engineering bacterium to study the TAG synthesis pathway [6, 18, 24, 38, 40]. For example, Cui et al. 7 showed that *HpDGAT2A*, *HpDGAT2B*, *HpDGAT2D* and *HpDGAT2E* were able to restore TAG synthesis in H1246 yeast with a large difference in enzymatic activity. These results demonstrate the remarkable role of H1246 yeast in the study of the TAG synthesis pathway and provide a solid basis for expressing the *DGAT* gene in yeast H1246 and studying its expression.

## Materials and methods

### *ClDGAT* gene identification

*C. lacryma-jobi* L. genome sequence was downloaded from the NCBI database (GenBank assembly accession: GCA_009758035.1). Arabidopsis *DGAT* genes were utilized as a reference for the identification of orthologous *ClDGAT* family genes. To identify *ClDGAT*s, particularly singleton genes, a hidden Markov model (HMM) profile was constructed from orthologous amino acid sequences and used as an HMM search query against *C. lacryma-jobi* L. gene models using the HMMER3 package. To identify additional *ClDGAT* genes, BLASTP was used for sequence similarity searches against a reference sequence protein database. ExPASy (https://web.expasy.org/protparam/) was used to compute the isoelectric point and molecular weight of putative *ClDGAT* proteins identified herein. The subcellular (http://linux1.softberry.com/) and CELLO (http://cello.life.nctu.edu.tw/) tools were used to predict the subcellular localization of proteins.

### Sequence similarity and phylogenetic analyses

The amino acid and CDS sequences of *DGATs* in a range of representative eukaryotic plants were downloaded from the NCBI (Table S1). These *DGAT* sequences and the *ClDGAT* sequences identified herein were aligned using ClusalW, then used to construct a neighbor-joining tree with 1000 bootstrap replicates using MEGA 10.

### Chromosomal distribution and collinearity analyses

The locations of putative *ClDGAT* genes within the *C. lacryma-jobi* L. genome were determined based on genomic sequence and annotation files, followed by mapping to appropriate chromosomes. Collinearity analyses were performed by comparing orthologous genes in *C. lacryma-jobi* L., *Zea mays* (GCF_902167145.1) and *Sorghum bicolor* (GCF_000003195.3_) with the TBtools MCScanX toolkit.

### Analyses of gene structure and conserved protein domains

NCBI CDD (https://www.ncbi.nlm.nih.gov/ Structure/cdd/wrpsb.cgi) and SMART (http://smart.embl-heidelberg.de/) were used to identify conserved protein domains. Conserved amino acid sequences for *ClDGAT*s were identified using MEME SUITE (minimum width ≥ 6, maximum width = 50). *ClDGAT* gene exon–intron structures were identified and displayed with the TBTool.The Transmembrane Prediction Tool (https://services.healthtech.dtu.dk/service.php?TMHMM-2.0) was used to predict transmembrane domains. DNAMAN tools were used for cluster members’ multiple sequence alignment and visualization of cluster members. SOPMA (http:///npsapbil.ibcp.fr/) was utilized to assess the inferred secondary structure. Tertiary structural predictions were made using SWISS-MODEL (https://swissmodel.expasy.org/). Screen models with 1 ≥ GMQE ≥ 0.8 and sequence similarity ≥ 75%.

### *ClDGAT*s’ CDS cloning and the construction of yeast expression vectors

The plant materials were obtained from the *Coix lacryma* germplasm resource nursery, which is located in the College of Life Science and Medicine, Zhejiang Sci-Tech University, Hangzhou, Zhejiang province, China. The *Coix lacryma* fruits were harvested for total RNA extraction. Extraction was performed using an RNA extraction kit (Vazyme, China). Use the HiScript lV All-in-One Ultra RT SuperMix for qPCR (Vazyme, China) to perform cDNA synthesis. Using the *ClDGATs* CDS sequences, the cloning primer were designed by the software CE Design (http://www.vazyme.com), as shown in Table S2. A total of ten *ClDGAT*s coding sequences originating from the plant materials were amplified by 2 × Phanta Flash Master Mix (Dye plus) P520 (Vazyme, China) using end-specific primers containing restriction sites.

### Yeast transformation and culture

In the yeast expression system, coding regions of ten *ClDGAT* genes were cloned into the yeast expression vector *pYes2*. *Saccharomyces cerevisiae* strain H1246 was transformed with ten constructed plasmids and empty vector control using the lithium acetate-mediated method. The *S. cerevisiae* strains H1246 used in this study, containing knockouts of all four neutral lipid biosynthesis genes *DGA1*, *LRO1*, *ARE1* and *ARE2*, was kindly provided by Professor Lixin Niu. All transformants were selected on the synthetic complete medium SC-Ura, supplemented with 2% (w/v) glucose and 2% (w/v) agar. The recombinant yeast cells were cultured in the 200 ml liquid medium SC-Ura, supplemented with 2% (w/v) galactose and 1% marshmallow. Induction culture was performed at a shaking table, 30 °C, 180 rpm in the dark. After induction culture for 5 d to reach the stationary phase. After centrifugation at 4000 rpm for 5 min to remove the culture solution, the harvested yeast was freeze-dried. Finally, the completely dried yeast was submerged in a grinder to be powdered for subsequent experiments.

### Quantitative RT–PCR

The relative transcript levels of *ClDGAT* genes were analyzed by RT-qPCR. RNAs were isolated from 2 d induced cultured yeast using RNA extraction kit (Vazyme, China). The reverse transcription reaction was carried out using HiScript lV All-in-One Ultra RT SuperMix for yeast (Vazyme, China). qPCR was performed using UniPeak U + One Step RT-qPCR SYBR Green Kit (Vazyme, China). The *Sc18S* was used as a reference gene, the template cDNA was diluted to 1/100. Thermal parameters for amplification were 95 °C for 30 s followed by 40 cycles of 95 °C for 10 s, 60 °C for 30 s. The expression levels of transcripts were calculated relative to the reference gene according to the equation: relative expression = 2–ΔCq, where ΔCq = Cq(target)-Cq(reference). Three technical replicates were performed for each sample. The oligonucleotide primer sequences were shown in Table S2.

### Lipid analysis

Weigh 10–20 mg (W1) of lyophilized yeast sample into a centrifuge tube. Add 1 mL of chloroform: methanol solution (2:1, v/v), mix thoroughly then leave it to stand. Add 0.5 mL of 0.9% (w/v) potassium chloride solution, mix well. Continue adding 1 mL of chloroform and mix well. Centrifuge at 4,000 rpm for 5 min to stratify the mixture. Transfer the lower chloroform phase out to a new centrifuge tube (W2) and blow it dry using a nitrogen purger. Weight the centrifuge tube again (W3). Add 100 μL of chloroform solution to dissolve the bottom grease completely for thin-layer chromatography (TLC) analysis. Transfer to a vial and stored at −20 ℃ for spare use. The total lipid content is calculated as follows: Total Oil Content = (W3 − W2)/W1.

Weigh out 50 mg of lyophilised yeast sample (W4) into a 50 ml centrifuge tube. Add 7.5 ml of a chloroform–methanol mixture (v:v = 1:2) and place in a shaking incubator at 37 °C and 200 rpm for 24 h. Centrifuge at 4,000 rpm for five minutes and collect the supernatant. Add another 7.5 ml of the chloroform–methanol mixture (v:v = 1:2) to the precipitate. Place in a shaking incubator at 37 °C and 200 rpm for 12 h. Centrifuge to collect the supernatant. Repeat this process once more, then combine the two resulting supernatants. Add 10 ml of a 1% NaCl solution and 5 ml of chloroform to the mixture, ensuring it is well mixed, then centrifuge at 4000 rpm for 5 min. Collect the lower chloroform phase into a pre-weighed 15 ml centrifuge tube (W5). Heat in a water bath until the organic phase evaporates and then dry in an oven. Finally, weigh the tubes containing fatty acids (W6). The total fatty acids content = (W5—W4)/W6.

The extraction experiment has three experimental replicates. The one-way ANOVA was used to analysis the data. And it was performed using SPSS softwear. The significance threshold was *P* > 0.001.

TLC was used to separate the lipid classes. A total of 20 μl chloroform extract were spotted onto the silica gel GF254 TLC plate (Haiyang, China). The plate was then placed in a chamber containing a mixture of hexane: ether: acetic acid (80:20:1, v/v/v). The TLC plates were stained in an iodine vat. After the color turns dark, photograph the TLC plates immediately.

### Nile Red staining

Aliquots of stationary phase cells (400 μl) were pelleted, washed twice in 1 × PBS, gently dispersed in 20 μl PBS, and mixed with 5 μl Nile Red (1 μg/μl) (Siloto et al. 2009). The stained cells were incubated in the dark for 10 min at 30 °C, then washed twice in PBS and diluted in 100 μl of 1 × PBS. The stained cells were observed and photographed under excitation light (wavelength between 560 and 620 nm) using a fluorescence microscope (Olympus IX71-A12FL/PH, Japan) equipped with a digital camera. Image-Pro Plus software (Media Cybernetics, Rockville, MD, USA) was used to analyze the fluorescence intensity of transgenic yeasts.

## Result

### Identification of *Coix lacryma-jobi *L. *DGAT* gene family members

*DGAT* family proteins have been identified in various plant species. To systematically identify *DGAT* genes within the *C. lacryma-jobi* L. genome, a hidden Markov model (HMM) and BLASTP method were used for whole-genome scanning. Following Pfam and Smart verification, redundant sequences in the annotated *C. lacryma-jobi* L. genome were removed. The remaining 10 putative *ClDGAT* genes identified via this approach were tentatively designated *ClDGAT1_1*, *ClDGAT1_2*, *ClDGAT1_3*, *ClDGAT2_1*, *ClDGAT2_2*, *ClDGAT3*, *ClWS/DGAT_1*, *ClWS/DGAT_2*, *ClWS/DGAT_3*, *ClWS/DGAT_4* based on their subfamilies. The predicted molecular weights (MWs) of these proteins ranged from 24 to 60 kDa, while their predicted isoelectric point (pI) values ranged from 4.9 to 10.5, indicating that these proteins were likely to be alkaline except for *ClWS/DGAT_1* and *ClWS/DGAT_3* (Table 1). Two online tools were used to predict the subcellular localization of the encoded proteins. It was shown that *ClDGAT1* and *ClDGAT2* uniformly localized in the plasma membrane. In contrast, *ClDGAT3* and different *WS/DGAT* proteins were predicted to be localized in diverse cellular compartments.

**Table 1:** Details of genome-wide identified *DGAT* family members in *C. lacryma-jobi* L

Accession numbers (ID)	Length (bp)	Name	Sub-family	Number of Amino Acid	Theoretical (pI)	Molecular Weight	Domain	Subcellular localization
Cl027022_T1	1383	*ClDGAT1_1*	DGAT1	460	9.04	52157.8	MBOAT superfamily	Plasma membrane
Cl031330_T1	1233	*ClDGAT1_2*	DGAT1	410	9.09	45566.35	MBOAT superfamily	Plasma membrane
Cl042438_T3	1344	*ClDGAT1_3*	DGAT1	447	9.56	48961.5	MBOAT superfamily	Plasma membrane
Cl018010_T2	1473	*ClDGAT2_1*	DGAT2	490	10.47	54371.36	PLN02783	Plasma membrane
Cl027481_T3	933	*ClDGAT2_2*	DGAT2	310	9.93	34847.86	PLN02783	Plasma membrane
Cl031870_T1	1260	*ClDGAT3*	DGAT3	419	7.38	44106.89	TRX_Fd_family	Chloroplast
Cl002154_T2	678	*ClWS/DGAT_1*	WS/DGAT2	225	4.91	24657.86	DUF1298	Peroxisome
Cl011297_T1	1506	*ClWS/DGAT_2*	WS/DGAT2	501	9.02	55161.62	DUF1298 and Condensation superfamily	Plasma membrane
Cl013404_T1	1566	*ClWS/DGAT_3*	WS/DGAT2	521	6.42	57385.44	DUF1298 and acyl_WS_DGAT superfamily	Mitochondrion
Cl041689_T1	1632	*ClWS/DGAT_4*	WS/DGAT2	543	9.37	60118.85	DUF1298 and acyl_WS_DGAT superfamily	Plasma membrane

**Fig. 2:**
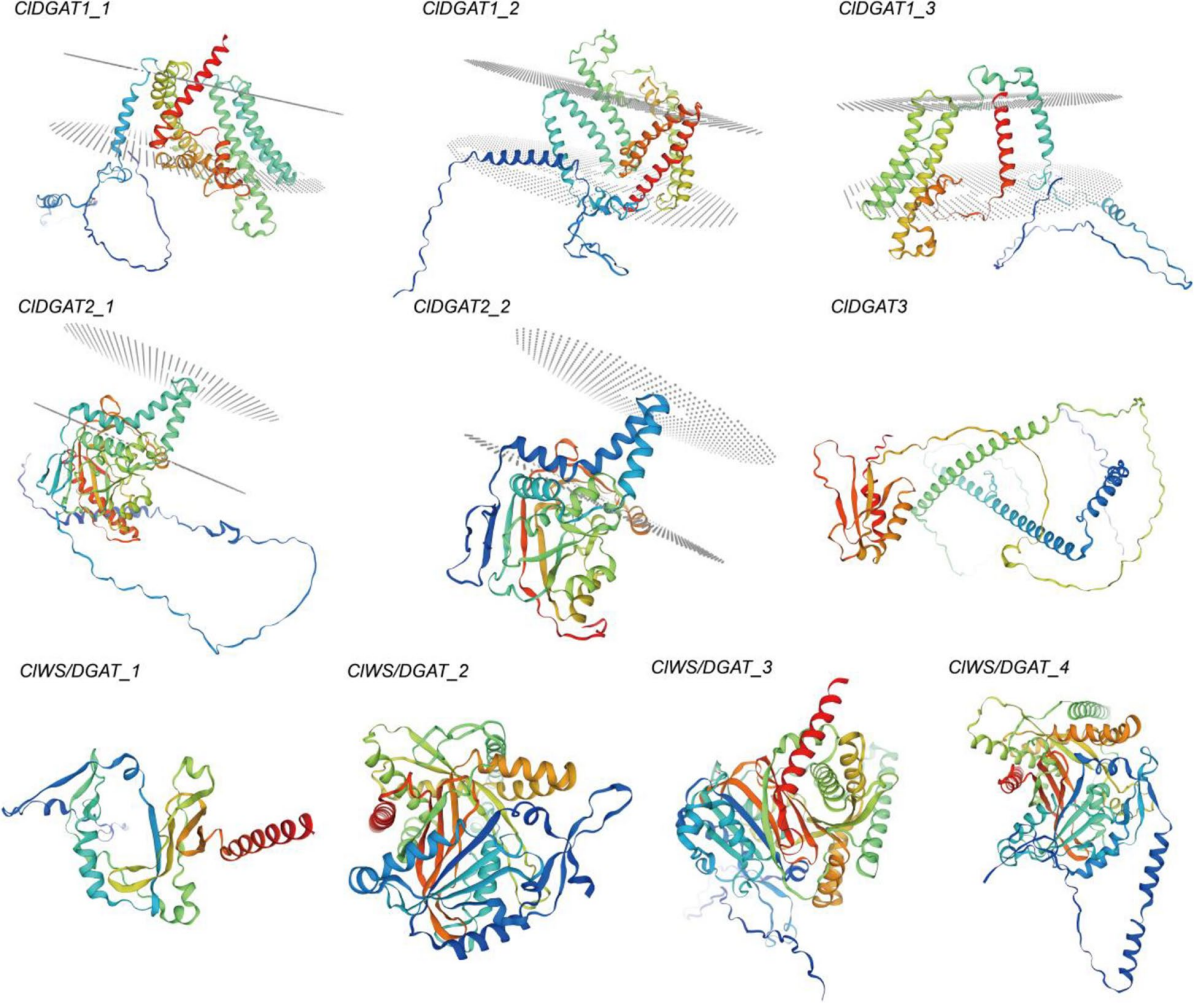
Protein structure prediction of ten *ClDGAT*s. The grey circular plane represents the cell membrane; Transmembrane domains: the peptide segments that traverse the cell membrane; α-helix: The helical structure; β-sheet: multiple parallel peptide segments

Meanwhile, the protein structures of these 10 *ClDGAT* genes were successfully predicted (Fig. 2). Both *ClDGAT1s* and *ClDGAT2s* contain several transmembrane domains, whereas *ClDGAT3* and *ClWS/DGAT* didn’t have any transmembrane domains. Based on the positive-inside rule, the N-terminus of *ClDGAT1s* and *ClDGAT2s* resided at the cytosolic side, and the C-terminus were pleased to the lumen side of the Endoplasmic Reticulum (ER). However, the protein structures of *ClDGAT*s from the same subfamily were very similar. Such as *ClDGAT1*s, they were characterized by the presence of 6 ~ 11 α-helix. Among them, *ClDGAT1-1* had 7 transmembrane helices and 2 intracellular loop; *ClDGAT1-2* had 8 transmembrane helix, 2 ER luminal (extracellular) loop and 2 intracellular loop; and *ClDGAT1-3* had 5 transmembrane helix, 1 ER luminal (extracellular) loop and 2 intracellular loop. *ClDGAT2-1* had 6 α-helices and 10 β-bends located on the cytosolic side of the membrane, as well as four transmembrane helixs; The structure of *ClDGAT2-2* revealed 5 α-helices and 9 β-bends on the cytosolic side of the membrane, as well as 4 transmembrane helices. *ClDGAT3* was quite different from the other *ClDGAT* family members. Its structure consisted of five α-helixes and four β-bends, with no transmembrane structure. As for the *ClWS/DGAT* subfamily, *ClWS/DGAT_1* was slightly different from other members, with only six α-helixes and seven β-bends which were not regular. However, the remaining three *ClWS/DGAT*s had similar structures, and they had 9 ~ 13 α-helixes and two sets of cross-parallel super-secondary structures consisting of 4 ~ 6 β-bends.

**Fig. 3:**
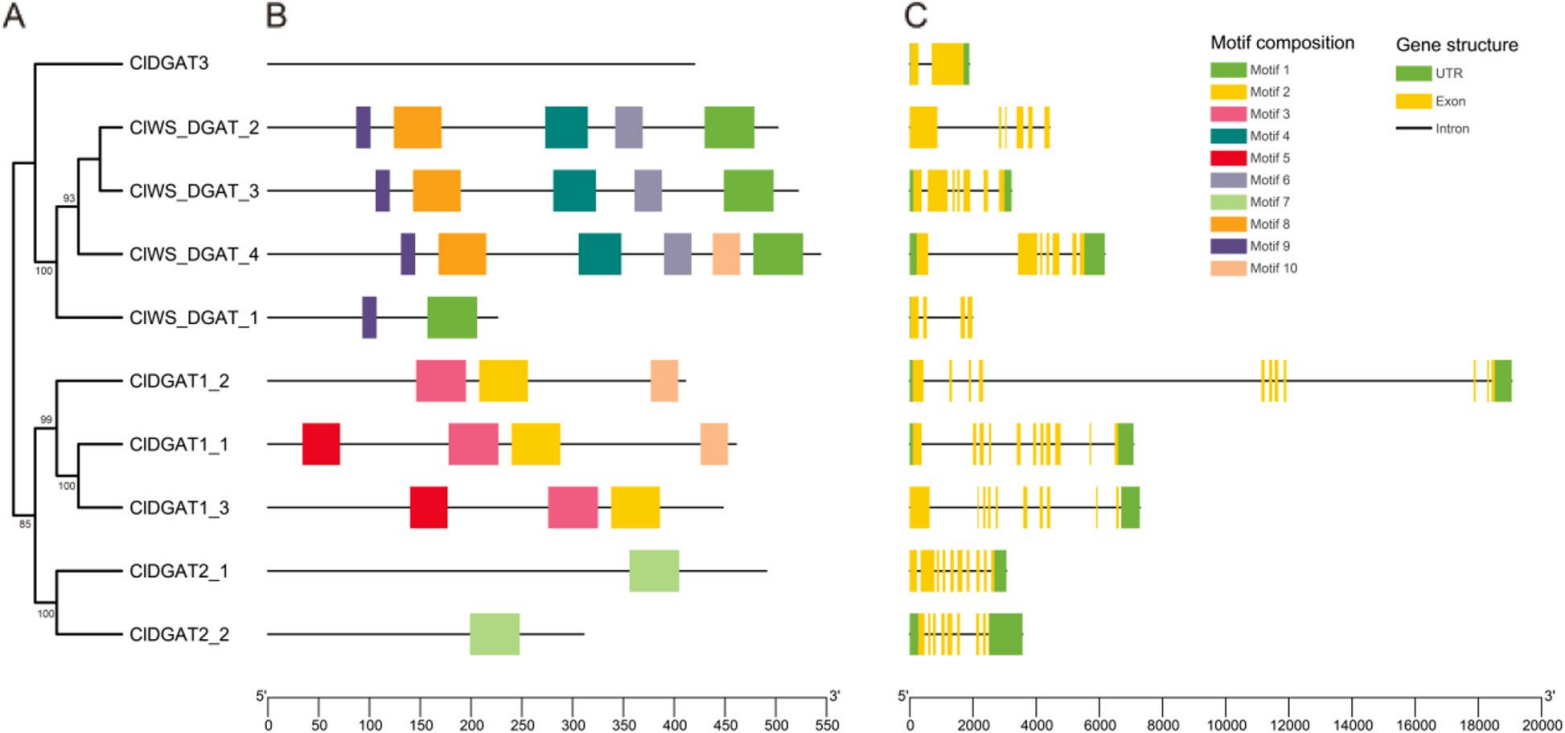
Gene motif analysis and gene structure demonstration. **A**. A neighbor-joining (NJ) phylogenetic tree of the *ClDGAT* family genes; **B**. Conserved structural domains of the *ClDGAT* family genes; **C**. Gene structure of the *ClDGAT* family genes

On the other hand, we also analyzed the conserved structural domains of the *ClDGAT* family genes (Fig. 3). Firstly, the motifs from different *ClDGAT*s were analyzed. The motifs from different subfamilies were quite different, while the motif of *ClDGAT3* was not even found. The *ClWS/DGAT* subfamily members had two conserved motifs, containing Motif 9 and Motif 1. The *ClDGAT1*s had a common motif 3 and motif 2, while *ClDGAT2* had a common motif 7, which predicted that they may have functional differences. Meanwhile, we found that *ClWS/DGAT_4*, *ClDGAT1_2*, and *ClDGAT1_1*, had a common Motif 10. This motif might enable them to have similar functions. Finally, the gene structure analysis of the *ClDGAT* gene family showed that *ClDGAT3* had only 2 exons, *ClWS/DGAT* had 4 ~ 7 exons, *ClDGAT1* had 10–11 exons, and *ClDGAT2* had 9–10 exons.

### Phylogenetic analysis of *ClDGAT* gene family members

**Fig. 4:**
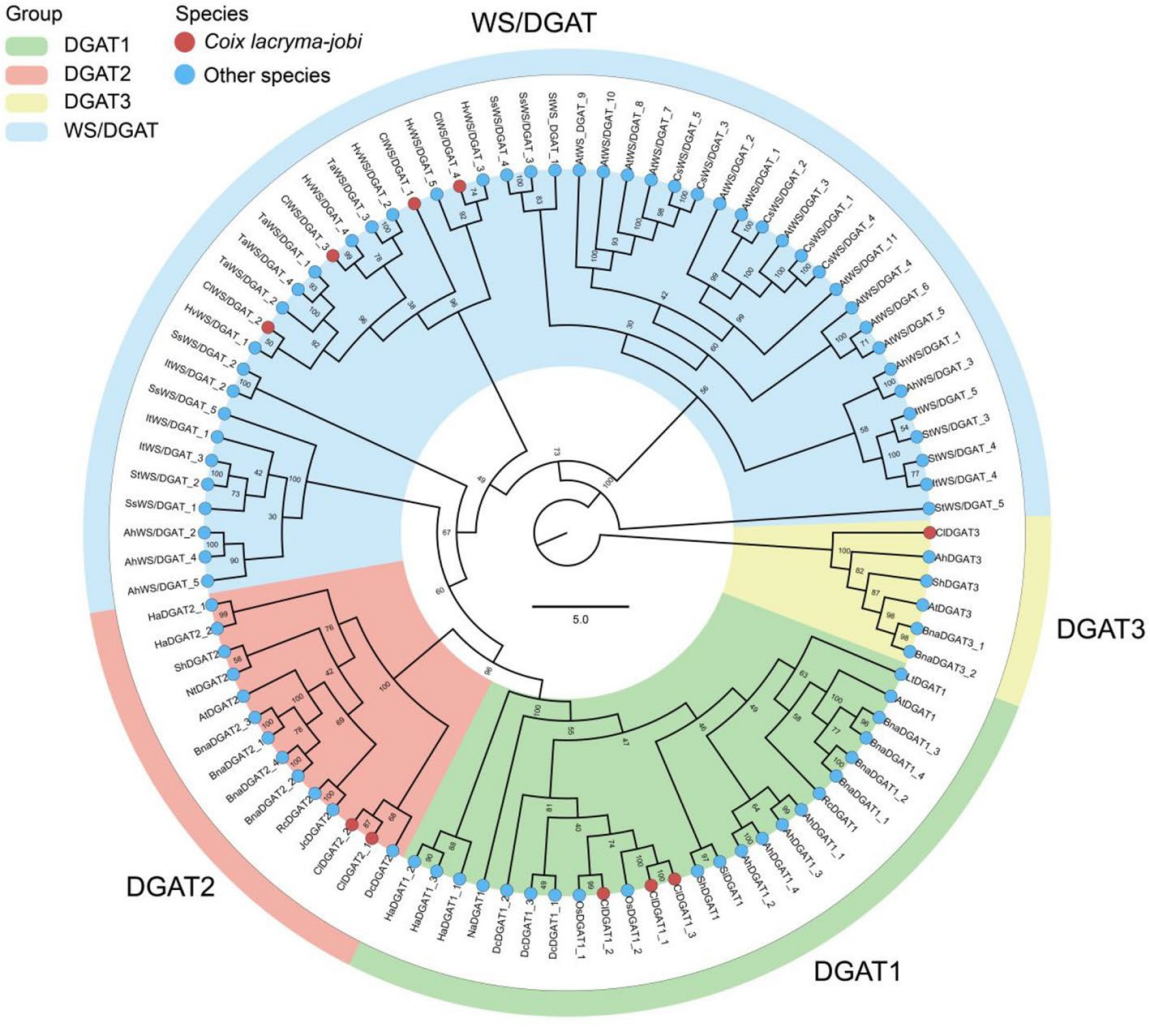
Phylogenetic analysis of *ClDGAT* gene family. A phylogenetic tree of *ClDGAT* proteins from *C. lacryma-jobi* L. and other plants was constructed by MEGA 10 using full-length protein sequences. Different branches were distinguished with different color shades. For the description of other species abbreviations involved in the figure, please see Additional Table S1

To understand the evolutionary relationships between *ClDGAT*s and related plant *DGAT*s, a neighbor-joining (NJ) phylogenetic tree incorporating the protein sequences for 70 *DGAT* genes across 19 representative eukaryotic species was constructed using MEGA 10. The description of those species abbreviations and details were listed in additional Table S1. A NJ phylogenetic tree was yielded in which the four major *DGAT* subfamilies were thoroughly separated, including *DGAT1*, *DGAT2*, *DGAT3*, and *WS/DGAT* clades (Fig. 4). As shown in Fig. 4, *ClDGAT1*, *ClDGAT2*, *ClDGAT3*, and *ClWS/DGAT* proteins respectively clustered with the *DGAT1*, *DGAT2*, *DGAT3*, and *WS/DGAT* clades. In the *DGAT1* class, all *ClDGAT1*s were clustered together with *Oryza sativa Japonica* (*OsDGAT1*), followed by the *Dendrobium catenatum1* (*DcDGAT1*) branches, suggesting that the *DGAT1* sequence of *C. lacryma-jobi* L. were more similar to *Oryza sativa* and *Dendrobium catenatum*. *ClDGAT2*s were similarly clustered together with *Dendrobium catenatum2* (*DcDGAT2*) s. *DGAT3*s were clustered separately, suggesting that it was relatively less similar to other *DGAT* family members. As for the *WS/DGAT* subfamily, all the *ClWS/DGAT*s were clustered together with *Hordeum vulgare subsp. vulgare* (*HvWS/DGAT*s), indicating that they had the highest similarity. In summary, *ClDGAT*s identified herein were more closely related to *Oryza sativa*, *Dendrobium catenatum*, and *Hordeum vulgare subsp DGAT*s, which shared the common feature of belonging to the Magnoliaceae but belonging to the Gramineae and Orchidaceae, respectively. In the other hand, this phenomenon predicted the close relationship between *C. lacryma-jobi* L. and those species.

### Chromosomal distribution and collinearity analyses of *ClDGAT* gene family members

**Fig. 5:**
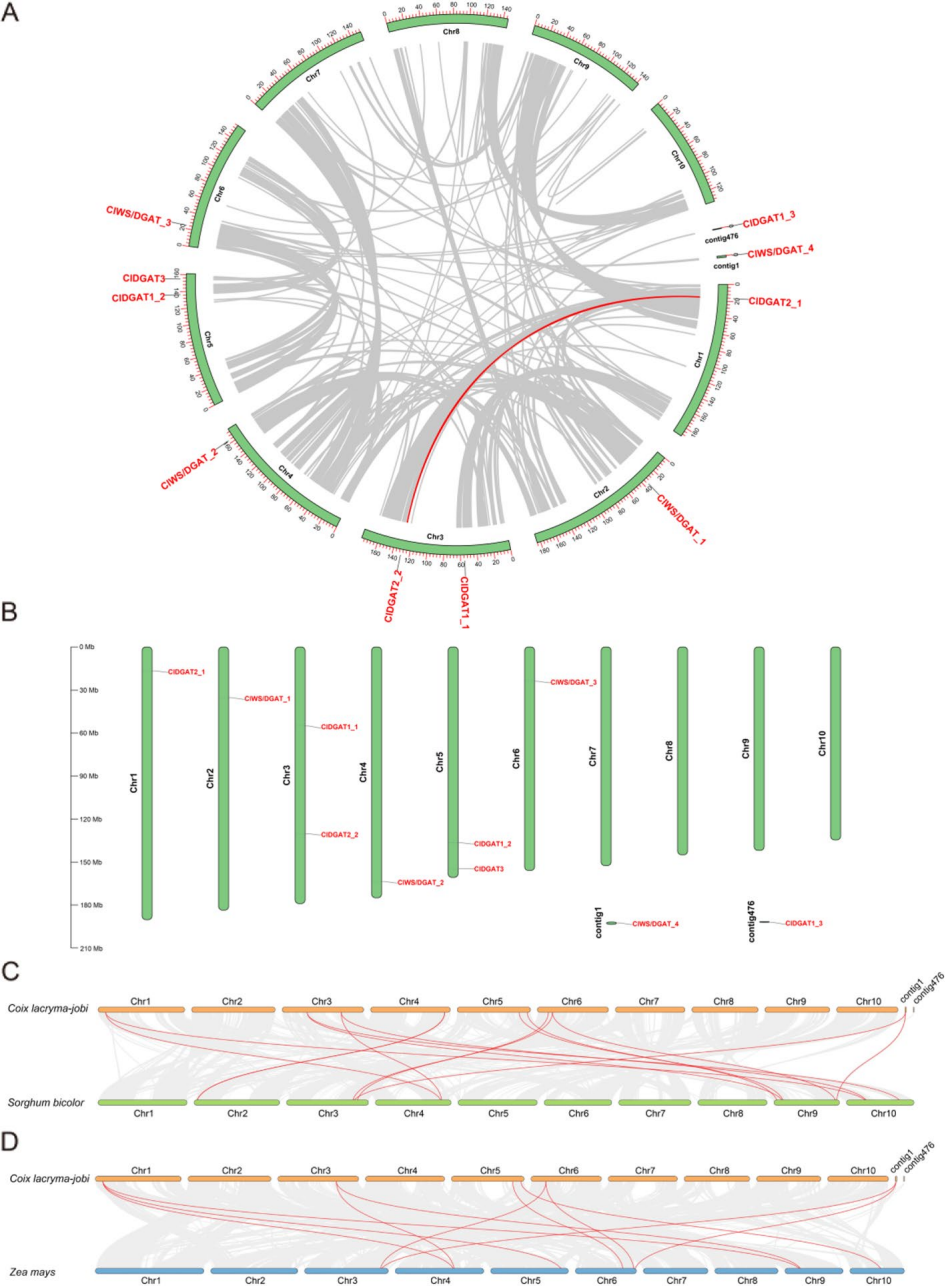
Chromosomal location and synteny analysis of *ClDGAT*s in *C. lacryma-jobi* L. genome. The scale is in megabases (Mb). The gray lines represent the collinear blocks within the genome, and the red lines highlight the syntenic pairs of DGAT genes. The number of chromosomes is displayed in the middle of each chromosome. **A**. Collinearity Analyses; **B**. Chromosomal locations of *ClDGAT*s. Tandem-duplicated genes are indicated with red color, and the chromosome number is indicated above each chromosome. **C**. Covariance analysis of *C. lacryma-jobi* with *Sorghum bicolor*; **D**. Covariance analysis of *C. lacryma-jobi* with *Zea mays*

The NCBI database was used to guide the mapping of these *ClDGAT* genes to the *C. lacryma-jobi* L. genome. As shown in the Fig. 5B, the *ClDGAT* genes unevenly distributed across ten *C. lacryma-jobi* L. chromosomes, with 1–2 genes per chromosome. Chromosomes 3, 5 encoded two of these *ClDGAT* genes, while chromosomes 1, 2, 4, and 6 only encoded a single *ClDGAT*, respectively. Notably, the number of *ClDGAT* genes per chromosome was not solely associated with chromosome size, what was given that the largest chromosome (Chr 1) only encoded one *ClDGAT*. In addition, two *ClDGAT*s were not successful compared to the chromosome. These findings demonstrated that *ClDGAT* family genes were not evenly distributed across the ten *C. lacryma-jobi* L. chromosomes.

Covariance allows the genes that are directly homologous between species to be arranged in a conserved order, which responds to the evolutionary relationship between species and the genomic position on the chromosomes. It has been shown in the literature that the *Sorghum bicolor* L. and *Zea mays* L. genome were most similar to *C. lacryma-jobi* genome [9, 37]. Therefore, we performed covariance analysis of *C. lacryma-jobi* with *Sorghum bicolor* and *Zea mays*, respectively (Fig. 5C, and D). It was found that there were 14 pairs of covariance between *C. lacryma-jobi* and *Sorghum bicolor*, and 11 pairs of covariance between the *DGAT* gene families of *C. lacryma-jobi* and *Zea mays*, exceeding the number of *ClDGAT* family members. It was suggested that these *ClDGAT*s were also highly conserved in the evolutionary process and might originate from a common ancestor of *Sorghum bicolor* and *Zea mays,* with similar *DGAT* functions. In comparison, *C. lacryma-jobi* and *Sorghum bicolor* were more closely related to each other. Among the covariate relationships within *C. lacryma-jobi* L. genome, only one covariate between two *ClDGAT* existed, suggesting that the rest *ClDGAT* members were not similar and conserved with each other (Fig. 5A).

### Overexpression of *ClDGATs* functionally restores oil biosynthesis in TAG-deficient mutants of yeast H1246

In summary, the structure of the *ClDGAT*s from different subfamilies were quite different and the sequence similarity varied considerably, so that the different *ClDGAT*s might have functional differences of lipid synthesis. In order to study their functional differences, we made them expressed in H1246 yeast to investigate their functions. Several studies had shown that expression of the *DGAT* gene in the yeast H1246 restores its oil production function [2, 16, 20]. For ease of presentation, we named the H1246 yeast lines containing the different *ClDGAT* genes as *ClDGAT1_1*, *ClDGAT1_2*, *ClDGAT1_3*, *ClDGAT2_1*, *ClDGAT2_2*, *ClDGAT3*, *ClWS/DGAT_1*, *ClWS/DGAT_2*, *ClWS/DGAT_3*, *ClWS/DGAT_4.* And the H1246 yeast lines containing no vector were named negative control. All strains had been verified by PCR (Fig. S2).

Firstly, a Nile red staining test was performed to identify lipid droplet formation. Nile red dye could excite orange fluorescence under blue light when combined with neutral lipid. In this study, a large number of lipid droplets were produced in the H1246 yeast expressing *ClDGAT* genes (Fig. S3). Therefore, we could conclude that TAG synthesis was restored in the strain H1246 transformed by the *ClDGAT* genes.

**Fig. 6:**
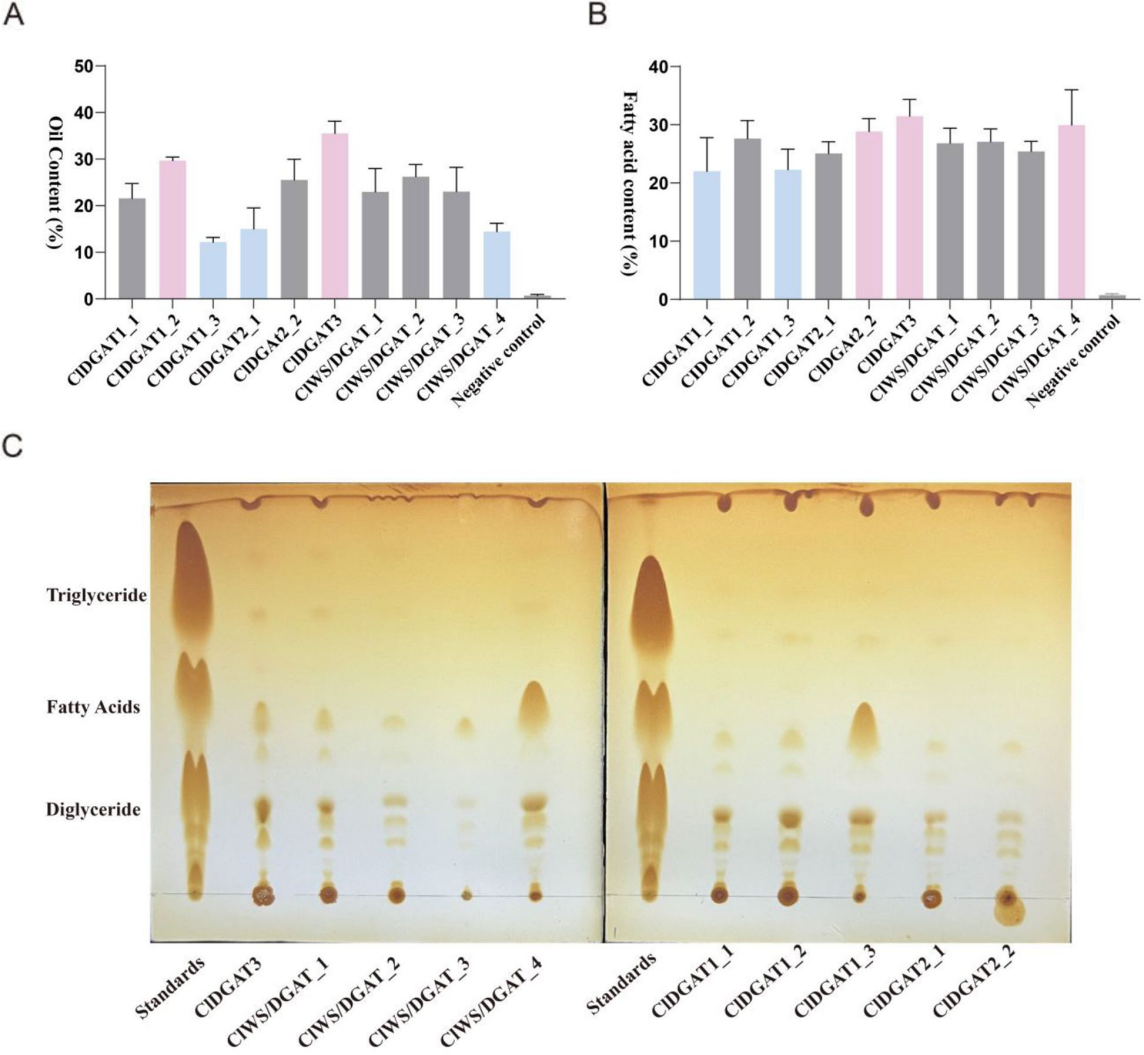
Analysis of oil and fatty acids synthesis ability. **A**. Total oil content of ten H1246 yeast lines. Oil content (%) was the total oil content generated in the samples. **B**. Total fatty acids content of ten H1246 yeast lines. Fatty acid content (%) was the quantification of total fatty acids in the samples. **C**. TLC test of ten H1246 yeast lines

In order to further investigate the function of synthetic lipids of different *ClDGAT*s, the genes expression, oil content and fatty acids content in the different H1246 yeast lines were tested. The one-way ANOVA analysis was used to analyze the significance of differences among different H1246 yeast lines (Table S3). After three days of induction culture, a quantitative Real-time PCR was performed to check the gene expression of the harvested yeast. The gene expression of 10 *DGAT* genes in negative control was almost undetectable, which afforded a reference to calculate the DGAT gene expression of corresponding H1246 lines. As shown in Fig. S4, the expression levels of *DGAT* genes were significantly high, indicating that each H1246 yeast line stably overexpressed the *ClDGAT* genes. Although ten H1246 yeast lines showed different gene expression levels on the third day of growth, they didn’t have significant differences, except of *ClDGAT2_2* (Fig. S4). Then, as shown in Fig. 6A and B, the oil and fatty acids content of the negative control were significantly lower than that of other yeast lines, indicating that the transference of *ClDGAT*s successfully restored the oil production capacity of H1246 yeast. The oil contents of *ClDGAT1_2* and *ClDGAT3* were significantly higher than that of the others. The oil content of *ClDGAT1_3*, *ClDGAT2_1* and *ClWS/DGAT_4*, however, was much lower. (Fig. 6A). In addition, the one-way ANOVA analysis of fatty content showed a significant difference (Table S3). As shown in Fig. 6B, *ClDGAT2_2*, *ClDGAT3*, and *ClWS/DGAT4* exhibited a higher level of fatty acid content, while *ClDGAT1_1*, *ClDGAT1_3* had a lower fatty acid content level. This phenomenon might be due to the fact that the total amounts of fatty acids in the H1246 yeast lines were similar. The difference laid in the limited affinity of *ClDGATs* for different fatty acids. Those results illustrated that ten *ClDGAT*s indeed had a significant difference in oil synthesis.

On the other hand, a TLC test was performed to separate the lipid classes. As shown in Fig. 6C, every *ClDGAT* separated triglyceride, fatty acids, and diglyceride. And the oil spots of the negative control were significantly lighter and even couldn't been seen (Fig. S5). A closer look would reveal that the size and the shade of the spots were different. The triglyceride shade of *ClDGAT3, ClDGAT2_1, ClDGAT1_2, ClDGAT1_3* was deeper than others. Combining their total oil content, it illustrated that *ClDGAT3* and *ClDGAT1_2* had the best oil and triglyceride synthesis ability. Although *ClDGAT2_1* and *ClDGAT1_3* had low total oil contents, they had a great ability of triglyceride synthesis. *ClWS/DGAT_4* and *ClDGAT1_3* had bigger size spots of fatty acids than others, suggesting that lots of fatty acids were in the free state. Combining the fatty acid content of *ClDGAT1_3* (low fatty acid content), it seemed that only a few fatty acids combined in triglyceride and diglyceride in strain *ClDGAT1_3* H1246 line. At the same time, *ClWS/DGAT_4* had a high fatty acid content. Although *ClWS/DGAT_4* appeared to promote the synthesis of free fatty acids, it was inefficient at utilizing fatty acids and synthesizing triglycerides.

In addition, to provide further insights into *ClDGAT*s’ function, the expression patterns of these genes in different *coix* tissues had been collected (Fig. S6). Based on the *coix* RNA-seq data from our lab [37], we found that the expressions of *ClDGAT1_3*, *ClWS/DGAT_1*, *ClWS/DGAT_2*, *ClWS/DGAT_3* and *ClWS/DGAT_4* were extremely low in all tissues, and *ClDGAT3* had the highest expression. *ClDGAT1_1* and *ClDGAT2_2* showed high expression in fruits relative to other parts. *ClDGAT1_2* exhibited high expression in stems. As for *ClDGAT3*, its expression levels were highest in leaves, followed by stems, roots, and fruits.

## Discussion

In this study, we conducted a detailed analysis of the *ClDGAT* gene family, covering various aspects such as gene family member identification, subcellular localization, protein structure, conserved structural domains and so on. Currently, the *DGAT* gene family has been characterized in multiple plant species, like *Perilla frutescens* and *Cannabis sativa* L. [34, 36]. The number of identified *DGAT* genes varied among different plant species, while the number of *WS/DGAT* genes was generally higher than other *DGAT* gene types. For the protein structure research, given that Wang et al. 28 had comprehensively revealed the protein structure of human diacylglycerol O-acyltransferase-1 (*hDGAT1*, Fig. S1), our study compared the structure of *hDGAT1* and *ClDGATs* to assess the reliability of these *ClDGAT* protein models. The results showed that *ClDGAT1s* were most similar to *hDGAT1*, while *ClDGAT2s* and *ClWS/DGATs* had additional β-bends compared to *hDGAT1*, indicating that the protein structure of *DGAT1* was relatively conserved. Additionally, the functional domains of *ClDGATs* were characterized, revealing conserved MBOAT (PF03062), PLN02783, TRX_Fd_family, and DUF1298 domains. These differences of domains might be related to active sites involved in substrate binding, catalytic activity, or other key regulatory functions.

Phylogenetic trees and gene collinearity analysis were commonly used to elucidate the evolution and phylogenetic relationships of homologous genes among different species and had been widely applied [25, 26]. As shown in phylogenetic trees result, *ClDGAT1* and *ClDGAT2* were closely clustered together with the *DGAT* genes from the same genus plant, while *ClDGAT3* and *ClWS/DGAT* were closely clustered with *DGAT* genes from different genus plants, indicating that *DGAT* genes evolved from different ancestral lineages, and this finding was supported by other studies [36]. In addition, we compared several different gene families of *C. lacryma-jobi* L. to identify species with high homology to *C. lacryma-jobi.* L. Zhai et al. 42 found that the auxin response factor from *C. lacryma-jobi* L. (*ClARF*) and the *sorghum ARF* exhibited the closest relationship, followed by *maize* and *rice*. Hua et al. 12 found that the Heat shock protein 20 from *C. lacryma-jobi* L. (*ClHSP20*) showed the highest homology with 36 HSP20 gene pairs from *wheat*, followed by *maize*, *sorghum*, *barley*, and *rice*. Combining the results, the homology between *C. lacryma-jobi* L. and *Poaceae* plants was relatively high. Through collinearity analysis, we found that only *ClDGAT2_1* and *ClDGAT2_2* exhibited collinearity, indicating functional redundancy between them. Not only in *C. lacryma-jobi* L., functional redundancy within the *DGAT* gene family had been reported in other plants, such as *Perilla frutescens* and *Cannabis sativa* [34, 36].

In order to investigate the functional redundancy of the *ClDGATs*, we compared the lipid production of H1246 yeast lines with different *ClDGAT*s and found that they indeed exhibited difference in lipid synthesis function. *ClDGAT1_2* and *ClDGAT3* demonstrated the greatest oil production efficiency. Furthermore, previous studies had shown that the overexpression of *PfDGAT2-2* or *PfDGAT3-1* could also significantly increases seed oil content in *Arabidopsis* [36].

*DGAT* gene expression level and patterns also varied in different plant species. In *C. lacryma-jobi* L., *DGAT3* gene expression levels were relatively high. Combining with its oil production efficiency, it was indicated that *DGAT3* played a key role in oil accumulation in *C. lacryma-jobi* L.. In addition, *CsDGAT3* expression levels were high in all tissues of *Cannabis sativa*, further indicating the role of *DGAT3* in tissue development [34]. In *Elaeis guineensis*, *EgDGAT3_2* expression was higher than other *DGAT*s in the mesocarp [26]. Overall, *DGAT3* exhibited high expression levels and strong oil synthesis capacity in various plant species. This phenomenon provided a direction for future improvements of the *DGAT* gene family and laid a solid foundation for enhancing the quality of oilseed crops.

## Conclusions

In this study, we systematically identified and characterized ten *ClDGAT* family genes in the *C. lacryma-jobi* L. genome. Phylogenetic analysis classified these genes into four subfamilies (*DGAT1*, *DGAT2*, *DGAT3*, and *WS/DGAT*). They had distinct structural features, such as transmembrane domains in *ClDGAT1s* and unique α-helix/β-sheet arrangements in *ClDGAT3* and *ClWS/DGAT* members. Evolutionary relationships revealed close homology with *Oryza sativa* and *Hordeum vulgare*, suggesting functional conservation within the *Poaceae* family.

Functional validation in the yeast H1246 demonstrated significant divergence in lipid synthesis capacity among *ClDGATs*. Notably, *ClDGAT3* and *ClDGAT1_2* exhibited best triglyceride production, highlighting their potential as key regulators of lipid accumulation in *C. lacryma-jobi* L. In contrast, *ClDGAT1_3* and *ClWS/DGAT_4* showed limited ability of lipid synthesis and fatty acid utilizations.

These findings deepen our understanding of the molecular mechanisms underlying lipid biosynthesis in *C. lacryma-jobi* L. and provided a foundation for future applications in metabolic engineering. Targeting *ClDGAT3* or *ClDGAT1_2* through genetic modification could enhance oil yield in plants and offer opportunities for pharmaceutical or nutraceutical industries.

## Supplementary Information

Additional file 1.

Additional file 2.

Additional file 3.

Additional file 4.

## Data Availability

All relevant data can be found within the manuscript and its supplementary data published onlin.

## Abbreviations

C. lacryma jobi L.: Coix lacryma jobi L.
DGAT: Diacylglycerol acyltransferase
WS/DGAT: Wax easter synthase/ Diacylglycerol acyltransferase
ClDGAT: Coix lacryma jobi L. Diacylglycerol acyltransferase
OsDGAT: Oryza sativa Diacylglycerol acyltransferase
DcDGAT: Dendrobium catenatum Diacylglycerol acyltransferase
HvWS/DGAT: Hordeum vulgare subsp WS/DGAT
PDAT: Phospholipid:diacylglycerol acyltransferase
DGA1: Diacylglycerol acyltransferase 1
LRO1: Phospholipid:diacylglycerol acyltransferase
ARE: Acyl-coA-sterol acyltransferase
TAG: Triacylglycerol
FAD: Fatty acid desaturase
ALA: α-Linolenic acid
NJ tree: Neighbor-joining phylogenetic tree
TLC: Thin-layer chromatography
