# Supplementary material for: Genome-wide analysis of DGAT gene family in Coix lacryma jobi L. and functional characterization in yeast H1246
Source: BMC Plant Biol. 2025 Nov 28;25:1660. doi: 10.1186/s12870-025-07648-7 (PMC12664205; doi:10.1186/s12870-025-07648-7)
Supplement: Supplementary file 2 — Additional file 2. [file 12870_2025_7648_MOESM2_ESM.docx]

**Table S1** Gene ID and their abbreviations.

| subfamily | gene ID | Origin of species | Gene abbreviations |
| --- | --- | --- | --- |
| DGAT1 | LOC106436339 | *Brassica napus* | *BnaDGAT1_1* |
|  | LOC106375527 | *Brassica napus* | *BnaDGAT1_2* |
|  | LOC106352950 | *Brassica napus* | *BnaDGAT1_3* |
|  | LOC106382435 | *Brassica napus* | *BnaDGAT1_4* |
|  | LOC112738290 | *Arachis hypogaea* | *AhDGAT1_1* |
|  | LOC112723654 | *Arachis hypogaea* | *AhDGAT1_2* |
|  | LOC112790365 | *Arachis hypogaea* | *AhDGAT1_3* |
|  | LOC112696987 | *Arachis hypogaea* | *AhDGAT1_4* |
|  | LOC110928453 | *Helianthus annuus* | *HaDGAT1_1* |
|  | LOC110893727 | *Helianthus annuus* | *HaDGAT1_1* |
|  | LOC110886978 | *Helianthus annuus* | *HaDGAT1_2* |
|  | LOC4338045 | *Oryza sativa Japonica Group* | *OsDGAT1_1* |
|  | LOC4341317 | *Oryza sativa Japonica Group* | *OsDGAT1_2* |
|  | DGAT1 | *Sesamum indicum* | *SiDGAT1* |
|  | LOC8272569 | *Ricinus communis* | *RcDGAT1* |
|  | LOC110107905 | *Dendrobium catenatum* | *DcDGAT1_1* |
|  | LOC110097309 | *Dendrobium catenatum* | *DcDGAT1_2* |
|  | LOC110099629 | *Dendrobium catenatum* | *DcDGAT1_3* |
|  | LOC109219417 | *Nicotiana attenuata* | *NaDGAT1* |
|  | LOC125194449 | *Salvia hispanica* | *ShDGAT1* |
| DGAT2 | LOC110912448 | *Helianthus annuus* | *HaDGAT2_1* |
|  | LOC110909196 | *Helianthus annuus* | *HaDGAT2_2* |
|  | LOC110110262 | *Dendrobium catenatum* | *DcDGAT2* |
|  | LOC125211455 | *Salvia hispanica* | *ShDGAT2* |
|  | LOC107779882 | *Nicotiana tabacum* | *NtDGAT2* |
|  | LOC8258757 | *Ricinus communis* | *RcDGAT2* |
|  | DGAT2 | *Jatropha curcas* | *JcDGAT2* |
|  | LTHEOB_2450 | *Lasiodiplodia theobromae* | *LtDGAT2* |
|  | BnaA.DGAT2.a | *Brassica napus* | *BnaDGAT2_1* |
|  | BnaA.DGAT2.b | *Brassica napus* | *BnaDGAT2_2* |
|  | BnaC.DGAT2.a | *Brassica napus* | *BnaDGAT2_3* |
|  | BnaC.DGAT2.b | *Brassica napus* | *BnaDGAT2_4* |
| DGAT3 | LOC112722709 | *Arachis hypogaea (peanut)* | *AhDGAT3* |
|  | LOC125196152 | *Salvia hispanica* | *ShDGAT3* |
|  | BnaA.DGAT3 | *Brassica napus* | *BnaDGAT3_1* |
|  | BnaC.DGAT3 | *Brassica napus* | *BnaDGAT3_2* |
| WS/DGAT | LOC123116652 | *Triticum aestivum* | *TaWS/DGAT_1* |
|  | LOC123097975 | *Triticum aestivum* | *TaWS/DGAT_2* |
|  | LOC123172288 | *Triticum aestivum* | *TaWS/DGAT_3* |
|  | LOC123112831 | *Triticum aestivum* | *TaWS/DGAT_4* |
|  | LOC123442392 | *Hordeum vulgare subsp. vulgare* | *HvWS/DGAT_1* |
|  | LOC123406073 | *Hordeum vulgare subsp. vulgare* | *HvWS/DGAT_2* |
|  | LOC123449726 | *Hordeum vulgare subsp. vulgare* | *HvWS/DGAT_3* |
|  | LOC123444517 | *Hordeum vulgare subsp. vulgare* | *HvWS/DGAT_4* |
|  | LOC123444117 | *Hordeum vulgare subsp. vulgare* | *HvWS/DGAT_5* |
|  | LOC121754064 | *Salvia splendens* | *SsWS/DGAT_1* |
|  | LOC121753951 | *Salvia splendens* | *SsWS/DGAT_2* |
|  | LOC121751373 | *Salvia splendens* | *SsWS/DGAT_3* |
|  | LOC121747791 | *Salvia splendens* | *SsWS/DGAT_4* |
|  | LOC121811347 | *Salvia splendens* | *SsWS/DGAT_5* |
|  | LOC116033983 | *Ipomoea triloba* | *IrWS/DGAT_1* |
|  | LOC116029219 | *Ipomoea triloba* | *IrWS/DGAT_2* |
|  | LOC116028847 | *Ipomoea triloba* | *IrWS/DGAT_3* |
|  | LOC116024391 | *Ipomoea triloba* | *IrWS/DGAT_4* |
|  | LOC116015436 | *Ipomoea triloba* | *IrWS/DGAT_5* |
|  | LOC104782348 | *Camelina sativa* | *CsWS/DGAT_1* |
|  | LOC104719346 | *Camelina sativa* | *CsWS/DGAT_2* |
|  | LOC104769483 | *Camelina sativa* | *CsWS/DGAT_3* |
|  | LOC104792712 | *Camelina sativa* | *CsWS/DGAT_4* |
|  | LOC104735261 | *Camelina sativa* | *CsWS/DGAT_5* |
|  | LOC112697417 | *Arachis hypogaea* | *AhWS/DGAT_1* |
|  | LOC112785116 | *Arachis hypogaea* | *AhWS/DGAT_2* |
|  | LOC112755332 | *Arachis hypogaea* | *AhWS/DGAT_3* |
|  | LOC112716702 | *Arachis hypogaea* | *AhWS/DGAT_4* |
|  | LOC114926152 | *Arachis hypogaea* | *AhWS/DGAT_5* |
|  | LOC102603179 | *Solanum tuberosum* | *StWS/DGAT_1* |
|  | LOC102602717 | *Solanum tuberosum* | *StWS/DGAT_2* |
|  | LOC102602674 | *Solanum tuberosum* | *StWS/DGAT_3* |
|  | LOC102597188 | *Solanum tuberosum* | *StWS/DGAT_4* |
|  | LOC102587207 | *Solanum tuberosum* | *StWS/DGAT_5* |

**Table S2** Primers used in this study.

| Gene Name | Primer | primer sequence (5’ to 3’) | application |  |
| --- | --- | --- | --- | --- |
| *ClDGAT1_1* | DGAT1_1-F | cttggtaccgagctcggatccATGGCCCCGCCCCCCTCC | gene cloning |  |
|  | DGAT1_1-R | gcggccgttactagtggatccCTATCTGCTTGCCTGGGCC |  |  |
| *ClDGAT1_2* | DGAT1_2-F | cttggtaccgagctcggatccATGGCGGACTCCGACGAC |  |  |
|  | DGAT1_2-R | gcggccgttactagtggatccTTATTTTGTCTTCTCAGTCCGGTTC |  |  |
| *ClDGAT1_3* | DGAT1_3-F | cttggtaccgagctcggatccATGAAACGAGACGAGACCGACA |  |  |
|  | DGAT1_3-R | cttggtaccgagctcggatccATGAAACGAGACGAGACCGACA |  |  |
| *ClDGAT2_1* | DGAT2_1-F | cttggtaccgagctcggatccATGCAGAAACCTGTCTCACACG |  |  |
|  | DGAT2_1-R | gcggccgttactagtggatccTCAAAGAACTCTTAACCGAAGATCAG |  |  |
| *ClDGAT2_2* | DGAT2_2-F | cttggtaccgagctcggatccATGGGCGCGGATGGCGGC |  |  |
|  | DGAT2_2-R | gcggccgttactagtggatccCTATGTTTGGACATTGGACCGA |  |  |
| *ClDGAT3* | DGAT3-F | cttggtaccgagctcggatccATGGAGCTCACCGGCGCC |  |  |
|  | DGAT3-R | gcggccgttactagtggatccCTAGATAGGCGTCATGCCAAGAT |  |  |
| *ClWS/DGAT_1* | WSDGAT2_1-F | cttggtaccgagctcggatccATGGCGGTCGCTGCCCAA |  |  |
|  | WSDGAT2_1-R | gcggccgttactagtggatccTTAAACCTTGCTGATGTTATTAGTTGTATC |  |  |
| *ClWS/DGAT_2* | WSDGAT2_2-F | cttggtaccgagctcggatccATGCATGCAACCAGCCATCT |  |  |
|  | WSDGAT2_2-R | gcggccgttactagtggatccTTAATCGTTTTTGGTTGACGCC |  |  |
| *ClWS/DGAT_3* | WSDGAT2_3-F | cttggtaccgagctcggatccATGGATGCGGCGGCAGCC |  |  |
|  | WSDGAT2_3-R | gcggccgttactagtggatccTTAATCATTTCTTTGATTCTTTTCAATG |  |  |
| *ClWS/DGAT_4* | WSDGAT2_4-F | cttggtaccgagctcggatccATGAATCCTGCCGCGACG |  |  |
|  | WSDGAT2_4-R | gcggccgttactagtggatccTCATCTTTTTGAAGCTGCCTGA |  |  |
| *ClDGAT1_1* | qDGAT1_1-F | TGCAGCCTCACTTTACCAGT | qRT-PCR | |
|  | qDGAT1_1-R | CAGATAGCACTGCTGAGTCG |  |  |
| *ClDGAT1_2* | qDGAT1_2-F | GACGCCATCTTCAAGCAGAG |  |  |
|  | qDGAT1_2-R | AGCAGCCTTCATCAGGTTCT |  |  |
| *ClDGAT1_3* | qDGAT1_3-F | GTGCTGCCTCACTTTACCAG |  |  |
|  | qDGAT1_3-R | CAGATAGCACTGCTGAGTCG |  |  |
| *ClDGAT2_1* | qDGAT2_1-F | GTTCGTGCCGCTCAATGATA |  |  |
|  | qDGAT2_1-R | TAGTCCTCCACGTGCAAAGT |  |  |
| *ClDGAT2_2* | qDGAT1_1-F | TGCAGTCTGGATGCCCTTTA |  |  |
|  | qDGAT1_1-R | ATGCATGGGTCTAGGGAAGG |  |  |
| *ClDGAT3* | qDGAT3-F | CTACAATTGCCGCACCAGAA |  |  |
|  | qDGAT3-R | TCTGCTGGTTGTGGATGTCT |  |  |
| *ClWS/DGAT_1* | qWSDGAT2_1-F | ATTGCACCCAGCATCTTTGG |  |  |
|  | qWSDGAT2_1-R | TCTGGAAATTGTGCGTCGTC |  |  |
| *ClWS/DGAT_2* | qWSDGAT2_2-F | AGTGTCTACGGGCTTCAACA |  |  |
|  | qWSDGAT2_2-R | GAAGTCGTCCAAGAGTTGGC |  |  |
| *ClWS/DGAT_3* | qWSDGAT2_3-F | ACTCAAGGCAGCTAGAGCAA |  |  |
|  | qWSDGAT2_3-R | AGACAACTGGGTGTCCACAA |  |  |
| *ClWS/DGAT_4* | qWSDGAT2_4-F | CCTGCTGAGAAGGTGGAGTT |  |  |
|  | qWSDGAT2_4-R | CAAGCTTGATGGAGTTCGCA |  |  |
| *Sc18S* | QSc18S-F | AACTCACCAGGTCCAGACACAATAAGG |  |  |
|  | QSc18S-F | AAGGTCTCGTTCGTTATCGCAATTAAGC |  |  |

**Table S3** ANOVA analysis of Oil content, Fatty acids content and DGAT genes expression

The detail was listed in the additional Excel “**Table S3**”.
