# Supplementary material for: Genome-wide analysis of DGAT gene family in Coix lacryma jobi L. and functional characterization in yeast H1246
Source: BMC Plant Biol. 2025 Nov 28;25:1660. doi: 10.1186/s12870-025-07648-7 (PMC12664205; doi:10.1186/s12870-025-07648-7)
Supplement: Supplementary file 4 — Additional file 4. [file 12870_2025_7648_MOESM4_ESM.docx]

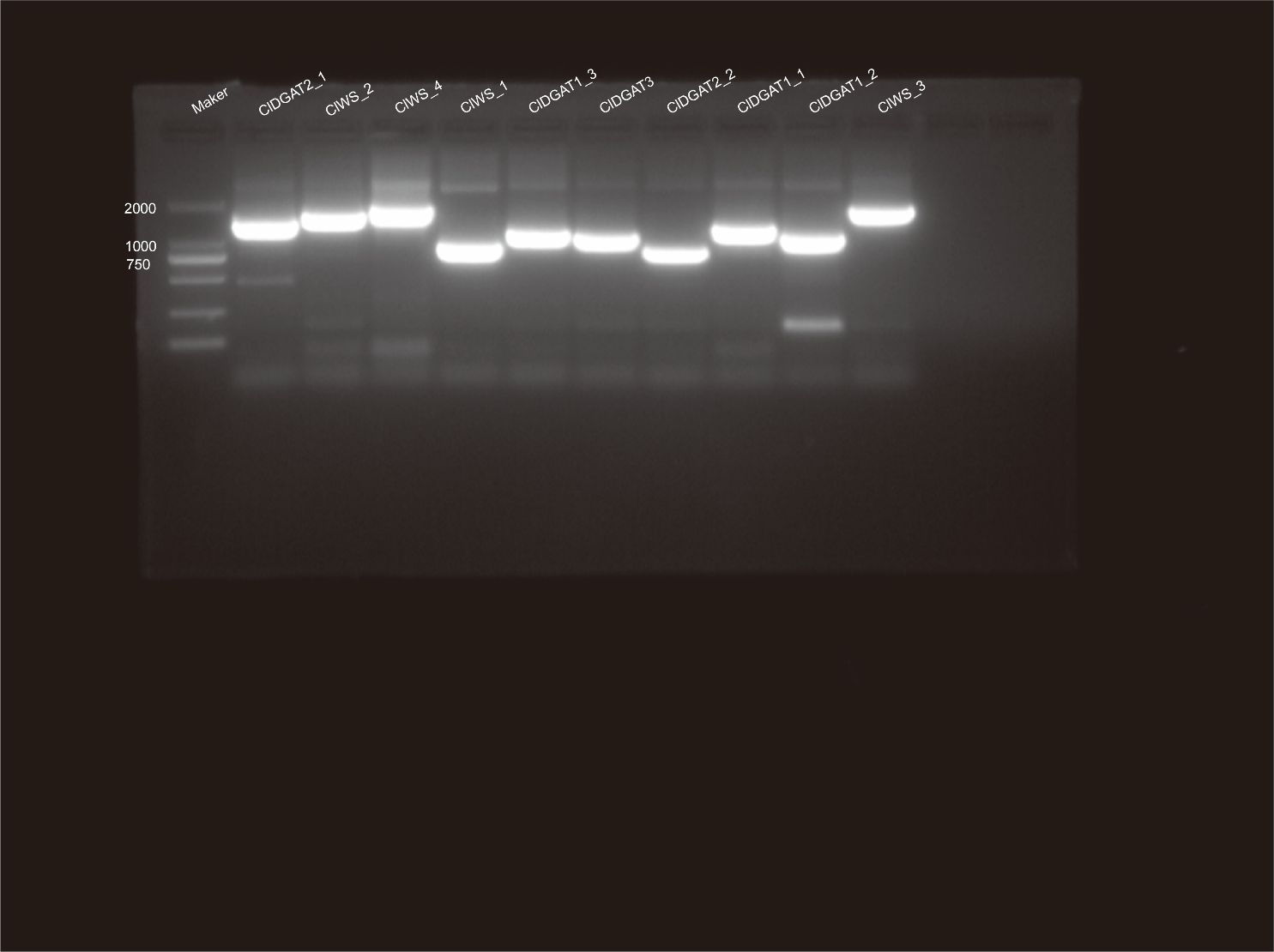
**unprocessed versions of all blots**

**Figure S2** Gel images showing double-digested fragments of the vectors. 2000-bp DNA marker. For the sake of visual appeal in the image, the *ClWSDGATs* have been abbreviated to *ClWSs*.
