# Supplementary material for: Genome-wide analysis of DGAT gene family in Coix lacryma jobi L. and functional characterization in yeast H1246
Source: BMC Plant Biol. 2025 Nov 28;25:1660. doi: 10.1186/s12870-025-07648-7 (PMC12664205; doi:10.1186/s12870-025-07648-7)
Supplement: Supplementary file 1 — Additional file 1. [file 12870_2025_7648_MOESM1_ESM.docx]

**Figure S1** The protein structure of human diacylglycerol O-acyltransferase-1 (*hDGAT1*)

**Figure S2** Gel images showing double-digested fragments of the vectors. 2000-bp DNA marker. For the sake of visual appeal in the image, the *ClWSDGATs* have been abbreviated to *ClWSs*.

**Figure S3** Nile red staining results of H1246 yeast containing different ClDGATs. The red image was detected under excitation light (The excitation wavelength between 560 and 620 nm), and the grey image was detected under bright field light.

**Figure S4** Quantitative DGAT genes expression analysis in transgenic yeast strain H1246. Reference genes were *S. cerevisiae* 18S Rrna. Values were shown as mean ± SD (n = 3). Set the expression levels of each gene in the negative control to 1 and calculate the gene expression levels in each H1246 line.

**Figure S5** TLC test of negative H1246 yeast lines. Negative control was the H1246 yeast without any vector *pYes2.*

**Figure S6** Expression patterns of *ClDGAT* genes in different tissues. R1, R2, R3 represent three biological replication of root samples. S1, S2, S3 represent three biological replications of stem samples. L1, L2, L3 represent three biological replications of leaf samples. G1, G2, G3 represent three biological replications of fruit samples.
